# Systemic Thrombolysis and Catheter-Based Therapies in Acute Pulmonary Embolism

**DOI:** 10.1016/j.jacadv.2024.101243

**Published:** 2024-09-12

**Authors:** Mariana Caetano Coelho, Rita Teixeira, Carla Nobre, Boban Thomas, Barbara Lacerda Teixeira

**Affiliations:** a Hospital Santa Marta, Lisbon, Portugal; b ULS Arco Ribeirinho, Setúbal, Portugal; c University Hospital Kerry, Tralee, Republic of Ireland; d University of California-San Diego, La Jolla, California, USA

**Keywords:** acute pulmonary embolism, chronic thromboembolic pulmonary hypertension, systemic thrombolysis

Already you know many things for yourself, and you will know others slowly.

— Pablo Neruda in Ode to Federico Garcia Llorca

We read with keen interest the Viewpoint by Rozenbaum regarding systemic thrombolysis (ST) in acute pulmonary embolism (PE) and disagree with the opinion expressed that the use of ST is becoming unethical because of low rates of major complications with catheter-based therapies (CBTs), such as percutaneous aspiration devices.^1^

There are no robust data supporting the use of CBTs in high-risk PE. In the FLowTriever for Acute Massive Pulmonary Embolism study in the “context arm” patients received diverse therapies, with a significant proportion (23%) of these high-risk PE patients being treated with anticoagulation alone, while being compared to the device.^2^

Although the FlowTriever All-Comer Registry for Patient Safety and Hemodynamics (FLASH) registry^3^ showed some promising results regarding the safety profile of one type of percutaneous aspiration devices, with low mortality and procedure-related complications, this study has limitations inherent to single-arm registries and is subject to many biases. Direct comparisons to other treatment options, especially ST, cannot be made, as only a minority (7.9%) of patients were considered high risk. Furthermore, the patient population in this registry was selected by each treating physician based on their judgment of suitability for mechanical thrombectomy, leading to potential selection bias and limiting the applicability of the results to the general PE population. Moreover, the characterization of the risk stratification is lacking.

Numerous options for CBTs for acute PE exist with none clearly superior to the other.^4^ Most of these devices do not have formal regulatory approval presently.

Primary percutaneous coronary intervention for ST-elevation myocardial infarction is not universally available throughout the world and ST is still used in resource-constrained and limited settings. Analogously, the widespread adoption of CBTs for acute PE worldwide is impractical and ST is a valuable resource. Reduced dose thrombolysis is being evaluated against standard anticoagulation in Pulmonary Embolism International THrOmbolysis (PEITHO)-3 study.
